# Intranasal Ectopic Tooth in Adult and Pediatric Patients: A Report of Two Cases

**DOI:** 10.1155/2019/8351825

**Published:** 2019-09-17

**Authors:** Isabela Polesi Bergamaschi, Bernardo Olsson, Aline Monise Sebastiani, Guilherme dos Santos Trento, Nelson Luis Barbosa Rebellato, Leandro Eduardo Klüppel, Delson Joao da Costa, Rafaela Scariot

**Affiliations:** ^1^Department of Stomatology, Department of Oral and Maxillofacial Surgery, Federal University of Paraná, Curitiba, Brazil; ^2^Cleft Lip and Palate Integral Care Center, Trabalhador Hospital, Curitiba, Brazil; ^3^School of Health Sciences, Department of Oral and Maxillofacial Surgery, Positivo University, Curitiba, Brazil

## Abstract

The aim of this study was to report two rare cases of ectopic tooth into the nasal cavity. The first case reports a 32-year-old female patient with the main complaint of having a tooth inside her nose. According to her, this condition causes pain and sporadic nosebleed. The patient had a facial trauma when she was 6 years old. The second case refers to an 8-year-old girl with left incomplete unilateral cleft lip and palate. The main complaint was left-sided nasal obstruction by a white hard mass. The treatment for both cases was surgical removal of the ectopic erupted tooth under general anesthesia. In conclusion, we can state that the surgical removal of intranasal tooth is a safe procedure and improves patient's quality of life.

## 1. Introduction

An aberrant tooth can be found in sites outside of the oral cavity and can be a supernumerary, deciduous, or permanent tooth [1]. The maxillary sinus and palate are the most frequently affected sites, while the mandibular condyle, coronoid process, orbits, and facial skin are affected much more rarely [1, 2]. The presence of supernumerary or ectopic teeth is not an uncommon fact, which occurs in 1% of the general population. However, the presence of teeth in the nasal cavity is a rare phenomenon, regardless of etiology [3]. The presence of teeth in the nasal cavity in cleft individuals is a rare phenomenon and obscure etiology and occurs in 0.1-1% of the general population [4]. Medeiros et al. found a prevalence of 0.48% intranasal teeth in children with complete cleft lip and palate [5].

The exact etiology of eruption of a tooth into the nasal cavity remains obscure. A few theories have been proposed to explain it, including the theory of developmental origin, which states that ectopic eruption may occur either due to reversion to the dentition of extinct primates having three pairs of incisor teeth, defect in migration of neural crest derivatives destined to reach the jaw bones, or due to a flaw in the multistep epithelial-mesenchymal interaction [6]. Other causes include developmental disturbances such as cleft lip and palate, trauma, or cystic lesions leading to tooth displacement, genetic factors, persistent deciduous teeth, and supernumerary teeth [7].

The purpose of this paper is to report two rare cases of ectopic tooth into the nasal cavity, with different etiologies, one caused by childhood trauma, while the other is associated with cleft lip and palate.

## 2. Case Report 1

A 32-year-old female patient, presenting normal facial growth, was referred to the Oral and Maxillofacial Surgery Service at Federal University of Paraná, Curitiba/PR, with the main complaint of having a tooth inside her nose. She reported this condition causes pain and sporadic nosebleed, especially on cold days. Patient's clinical history revealed that she had a facial trauma when she was six years old, affecting her superior anterior teeth, which was the etiological factor of the ectopic position of the tooth.

She had no history of systemic diseases or previous surgical procedures, although she was allergic to cephalexin. On clinical examination, raising the nasal tip, it was possible to observe the crown of the maxillary central incisor inside the right nostril.

**Figure 1:**
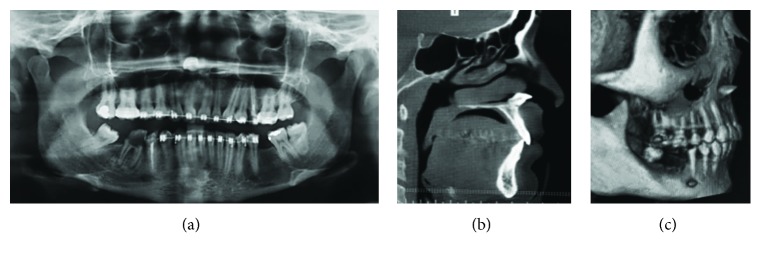
(a) Panoramic radiograph shows an ectopic tooth in the nasal cavity. (b) Sagittal cut of CT scan showing the horizontal orientation of the right superior incisor tooth inside the nasal nostril. (c) 3D reconstruction of CT scan showing the ectopic position of element 11.

The patient was submitted to a panoramic radiography, which showed the presence of a radiopaque mass similar to an incisor tooth on the right nasal cavity. For detailed radiographic examination, a computed tomography (CT) was requested and a high-density area was found located in a nasal nostril, horizontally arranged, incomplete rhizogenesis, with the crown facing to the exit of the nasal cavity (Figure 1).

**Figure 2:**
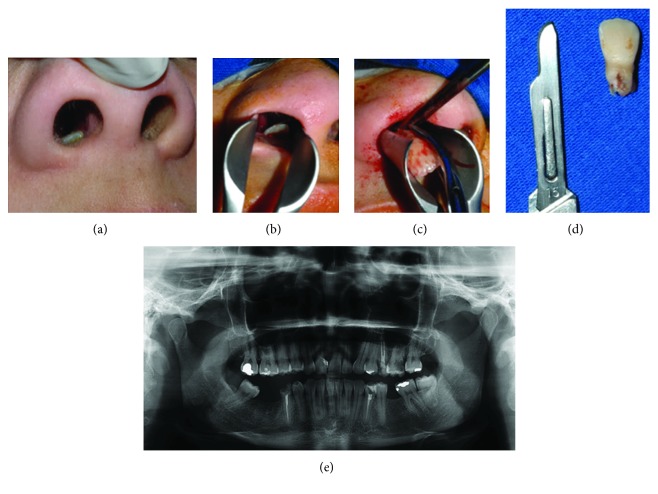
(a) Preoperative picture showing the exposition of the ectopic tooth inside the right nostril. (b, c) With the aid of nasal speculum, the tooth was removed by intranasal approach. (d) Tooth removed. (e) Panoramic radiograph confirms the complete removal of the element 11.

The surgical removal of the ectopic tooth was performed under general anesthesia, mainly because of the potential transoperative bleeding. After infiltration of local anesthesia with a vasoconstrictor, the tooth was removed using an intranasal approach with the aid of nasal speculum. Succeeding the extraction, nasal packing was placed to prevent postoperative epistaxis, which was removed 12 hours later, with no complications (Figure 2).

One-year follow-up shows resolution of the patient's main complaint, who reports a better breathing, no more nosebleed, or bad smells.

## 3. Case Report 2

An 8-year-old girl with left incomplete unilateral cleft lip and palate was referred to the Cleft Lip and Palate Integral Care Center (CAIF) with left-sided nasal obstruction due to an intranasal hard white mass surrounded by granulation tissue. The patient had no pain or any other complaints regarding the white mass, but because it was hypothesized to be an ectopic eruption of a dysmorphic lateral incisor with no possibility of orthodontic traction, the patient was advised to surgically remove the dysmorphic tooth. Intraoral examination and CT scan assured the hypothesis of ectopic eruption since the left lateral incisor was missing and the radiopacity of the mass matched the radiopacity of other teeth. The patient's surgical history was cheiloplasty at 4 months old, palatoplasty at 1 year old, microotological surgery and tonsillectomy at 5 years old, septoplasty at 6 years old, adenoidectomy at 7 years old, and alveoloplasty and autogenous iliac crest bone craft for oronasal fistula repair at 8 years old. No comorbidities were elicited.

**Figure 3:**
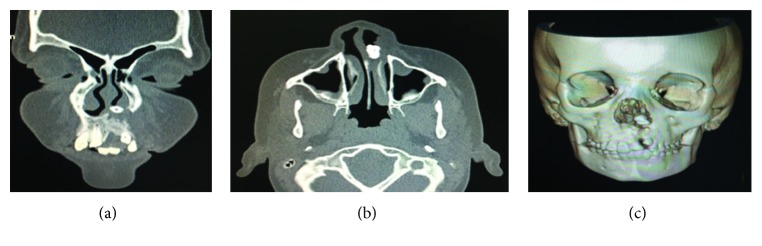
(a, b) CT scan showing intranasal location and soft tissue surrounding the dysmorphic tooth. (c) 3D reconstruction of CT scan showing the ectopic position of element 21.

CT scan revealed a radiopaque mass situated in the middle of the palatal cleft towards the left nasal cavity. Despite the superior portion, the dysmorphic lateral incisor was inserted in soft tissue (Figure 3). The tooth removal surgery was under general anesthesia because the patient was not cooperative.

**Figure 4:**
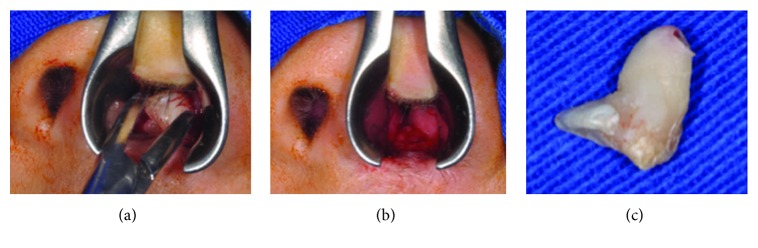
(a) Nasal speculum provided direct access to the toothlike structure removal with a Kelly hemostatic forceps. (b) Hemostasis obtained after the toothlike structure has been removed. No stiches were needed. (c) Ectopic tooth removed.

Because the dysmorphic tooth had no bone insertion and could be accessed using a nasal speculum, Kelly hemostatic forceps were chosen to remove the tooth (Figure 4). Hemostasis was obtained only by gauze pressure for a few seconds; no stiches were needed. Findings by nude eye examination confirmed a 10 millimeter wide toothlike structure (Figure 4). On two weeks of follow-up and postsurgery CT scan examination, the patient still had no complaints and the left airway was normal compared to the opposite side, respectively. One year follow-up and the patient still has no complaint.

## 4. Discussion

Intranasal tooth represents a small portion of all reported cases of ectopic eruption in the literature. Although the occurrence of supernumerary teeth is rare, up to 1% [4], it overcomes the prevalence of deciduous and permanent teeth found in the nasal cavity [2, 8]. In this report, we show two rare cases of permanent teeth found intranasally, one in a 32-year-old woman and the other in an 8-year-old girl. The age range reported for detecting the nasal tooth is broad, from 3 to 62 years [7, 9]. This range is broad due to different conditions that lead the patient to seek treatment [1]. The two cases in this report are examples of conditions: early detection happened because the patient was under treatment for cleft lip and palate, whereas late detection, at 32 years, happened due to professional misinformation provided to the patient, leading her to treat only the symptoms, not the cause of pain and nasal bleeding.

Both cases agree with previous reports regarding no predisposition for left or right nostril [7], no occurrence in both nostrils [2], and symptoms such as nasal obstruction and epistaxis [2, 8]. The lack in pattern for left or right nostril could be explained by the possible etiology of the ectopic eruption: obstruction by the time of eruption caused by persistent deciduous teeth; no space in the arc; intrusive luxation; facial deformities, such as cleft palate; cysts; and genetic predisposition [5]. Despite crowded dentition, trauma (i.e., intrusive luxation) [10], and facial deformities (i.e., cleft palate [5, 11]), the other possible etiologies do not have a predilection for left, right, or anterior region.

The 32-year-old woman reported a traumatic injury involving her teeth when she was 6 years old, age when the front incisor are in eruption route [6] and Nolla stage 7 (complete crown and 1/3 of the root's development) [12]. On the other hand, the 8-year-old girl's mother said that the white mass appeared after 3 months her daughter had been through iliac crest bone craft for oronasal fistula repair. Although both conditions are different, both are capable of causing tooth displacement [6, 13]. Since cleft palate has a multifactorial etiology, including environmental and genetic factors [11], genetic predisposition as an etiology for the ectopic eruption of the dysmorphic tooth in the case 2 cannot be neglected. We did not have access to previous CT scans or other radiographic images to determine whether or not trauma and maxilla crafting were the main causes of tooth ectopic eruption. Despite the lack of previous CT scans or other radiographic images, the patients' age suggests that Nolla stage in both of them could be classified as 7 or 8 [12], which could be a facilitating factor for the tooth displacement.

To accomplish a precise diagnosis in both cases, CT scans were requested for both patients in order to make a differential diagnosis between tooth in the nasal cavity, benign tumors, rhinolith [4, 6, 9, 14], and calcified inflammatory lesions due to tuberculosis and syphilis [9]. These hypotheses were negated by matching clinical and tomographic findings [2]. Another hypothesis that could be taken into consideration is an ectopic eruption of a supernumerary tooth [1]. Theories have been developed to explain the formation of a supernumerary tooth [1], including theories involving single nucleotide polymorphisms [15] and dental lamina hyperactivity [1, 16]. Clinically, both patients had missing permanent tooth. Tomographic examination showed matching radiopacity between normal teeth and the investigated teeth [2, 14]. Although histological examination has been performed by other authors to confirm the diagnosis of a tooth in the nasal cavity [6], the authors did not find it relevant as a method for differential diagnosis since both patients had a history of a missing permanent tooth in the oral cavity related to trauma or tissue manipulation in the area.

To solve symptoms and prevent further complications, surgically removing the tooth has been proposed as treatment [2, 6, 9, 14]. When the tooth is not inserted in the bone, the procedure becomes simple, but it still has important possible major complications, such as infection [17] and hemorrhage [18], that indicate general anesthesia as a safer protocol to be followed [17, 18].

Finally, intranasal ectopic tooth is somehow rare but is potentially harmful when left untreated; thus, surgically removing the intranasal ectopic tooth is important to improve patient's quality of life. Furthermore, the diagnosis is simple, fast, and cheap.
